# Iodine and Selenium Biofortification with Additional Application of Salicylic Acid Affects Yield, Selected Molecular Parameters and Chemical Composition of Lettuce Plants (*Lactuca sativa* L. var. *capitata*)

**DOI:** 10.3389/fpls.2016.01553

**Published:** 2016-10-18

**Authors:** Sylwester Smoleń, Iwona Kowalska, Małgorzata Czernicka, Mariya Halka, Kinga Kęska, Włodzimierz Sady

**Affiliations:** ^1^Unit of Plant Nutrition, Institute of Plant Biology and Biotechnology, Faculty of Biotechnology and Horticulture, University of Agriculture in KrakowKraków, Poland; ^2^Unit of Genetics, Plant Breeding and Seed Science, Institute of Plant Biology and Biotechnology, Faculty of Biotechnology and Horticulture, University of Agriculture in KrakowKraków, Poland

**Keywords:** beneficial elements, biofortification, iodine, salicylic acid, selenium, selenocysteine, selenocysteine methyltransferase (*SMT*), selenomethionine

## Abstract

Iodine (I) and selenium (Se) are included in the group of beneficial elements. They both play important roles in humans and other animals, particularly in the regulation of thyroid functioning. A substantial percentage of people around the world suffer from health disorders related to the deficiency of these elements in the diet. Salicylic acid (SA) is a compound similar to phytohormones and is known to improve the efficiency of I biofortification of plants. The influence of SA on Se enrichment of plants has not, however, been recognized together with its effect on simultaneous application of I and Se to plants. Two-year studies (2014–2015) were conducted in a greenhouse with hydroponic cultivation of lettuce in an NFT (nutrient film technique) system. They included the application of I (as KIO_3_), Se (as Na_2_SeO_3_) and SA into the nutrient solution. KIO_3_ was used at a dose of 5 mg I⋅dm^-3^ (i.e., 39.4 μM I), while Na_2_SeO_3_ was 0.5 mg Se⋅dm^-3^ (i.e., 6.3 μM Se). SA was introduced at three doses: 0.1, 1.0, and 10.0 mg⋅dm^-3^ nutrient solutions, equivalent to 0.724, 7.24, and 72.4 μM SA, respectively. The tested combinations were as follows: (1) control, (2) I + Se, (3) I + Se + 0.1 mg SA⋅dm^-3^, (4) I + Se + 1.0 mg SA⋅dm^-3^ and (5) I + Se + 10.0 mg SA⋅dm^-3^. The applied treatments had no significant impact on lettuce biomass (leaves and roots). Depending on the dose, a diverse influence of SA was noted with respect to the efficiency of I and Se biofortification; chemical composition of leaves; and mineral nutrition of lettuce plants, including the content of macro- and microelements and selenocysteine methyltransferase (*SMT*) gene expression. SA application at all tested doses comparably increased the level of selenomethionine (SeMet) and decreased the content of SA in leaves.

## Introduction

### Mineral Nutrients – I and Se

A widespread problem around the world is the deficiency of mineral nutrients and vitamins in people and farm animals. The most common are the deficiencies of Fe, Zn, I, and Se, affecting 60, 30, 30, and 15% of the population, respectively. The main cause of insufficient content of mineral nutrients in food is low concentration of its available forms in soils. This subsequently affects poor transfer of macro-, micro- and beneficial elements in the soil–plant–consumer system (Hirschi, 2009; White and Broadley, 2009; Wu et al., 2015).

This problem has intensified due to the cultivation of high-yielding cultivars that were introduced into agricultural practice as an effect of the ‘Green Revolution.’ These genotypes, particularly hybrids, are characterized by their lower content of mineral nutrients compared to traditional genotypes (Fan et al., 2008; Anandan et al., 2011). For this reason, it is suggested to introduce plant biofortification by agrotechnical or biotechnological approaches in order to improve, among other things, the accumulation of mineral nutrients in food and fodder (Hirschi, 2009; White and Broadley, 2009). Another important issue is the implementation of the ‘Second Green Revolution,’ the goal of which would be to improve crop yield and increase the transfer of mineral nutrients from soils to the food chain (Lynch, 2007). It needs to be remembered that the agrotechnical method of plant biofortification with nutrients is a relatively low-cost approach to the prophylaxis of nutrient deficiency of consumers (Cakmak, 2008; White and Broadley, 2009; Zhao and McGrath, 2009; Przybysz et al., 2015).

The need for conducting simultaneous plant biofortification with I and Se is dictated by the crucial role that both elements play in functioning of the thyroid gland, which, through respective hormones, ensures the proper condition of an entire organism. The basic relationship between I and Se in humans (and other animals) is that three of the iodothyronine deiodinases (D1, D2, D3) are Se-dependent enzymes (Bianco and Kim, 2006).

Thus far, I prophylaxis in many countries throughout the world has been based on salt iodisation. Despite the excessive consumption of table salt (greater than the recommended amount by the World Health Organization (WHO) of 5 g NaCl⋅day^-1^), the problem of I deficiency has not been solved. The WHO recommends developing alternative methods of I introduction into the diet. However, the implementation of I biofortification of plants was not reviewed by WHO experts (WHO, 2004, 2014). In our opinion, the WHO perspective of that matter needs to change. Studies conducted on both plants and rats so far have demonstrated that those biofortified with I are a sufficient or even better source of this micronutrient than those biofortified with KI (Tonacchera et al., 2013; Kopeć et al., 2015; Piątkowska et al., 2016). It also was shown that extracts from I-enriched plants may inhibit Caco-2 cancer cell proliferation as opposed to using KI (Koronowicz et al., 2016).

For several years in Finland and Malawi, nationwide agrotechnical programs of crop fertilization/biofortification with Se have been conducted (Eurola et al., 2003; Chilimba et al., 2012). In Xinjiang Province, China, enrichment of water used for watering fields with KIO_3_ was carried out (Ren et al., 2008). In comparison to global needs, these actions leading to the increase of I and Se levels in soils are insufficient.

The main factor limiting the introduction of agrotechnical methods of plant biofortification with I and Se is that both of these elements are not required plant nutrients (Kopsell and Kopsell, 2007; Kabata-Pendias, 2011). For several dozen years, Se has been classified as a beneficial element for plants. It has been postulated that I also should be included in this group, as there is information pointing to its positive effects on N use efficiency by plants (Blasco et al., 2012; Smoleń and Sady, 2012) and nutritional quality of tomato fruits (Kiferle et al., 2013; Smoleń et al., 2015). It raises the need to carry out basic research focussed on the assessment of simultaneous application of I and Se to plants; however, these issues are poorly documented. Only a few studies have been conducted thus far regarding this aspect, including hydroponic cultivation of spinach (Zhu et al., 2004) and lettuce (Smoleń et al., 2014). More research, however, has been conducted on agrotechnical methods of plant biofortification/fertilization with individual applications of I (Blasco et al., 2010; Kato et al., 2013; Lawson et al., 2015) or Se (Ríos et al., 2008, 2010; Hawrylak-Nowak et al., 2015; Hernández-Castro et al., 2015).

### SA – Role in Plants and Possible Impact on Biofortification Process

There are different classifications of the physiological role of SA in plants. Some authors consider SA a phytohormone-like compound, as it is involved in the regulation of plant growth, development and other physiological processes (Fariduddin et al., 2003; Hayat et al., 2010). For others, it is even included in the group of phytohormones (Gust and Nürnberger, 2012).

It is worth mentioning that SA is engaged in plant adaptation/protection from biotic and abiotic stress factors (Hayat et al., 2010). Induction of plant systemic resistance relies on the conversion and volatilisation of the ester form of SA, methyl salicylate (MeSA). Natural pathways of the degradation of endo- and exogenous SA in plants involve SA volatilisation and formation of sugar conjugates with SA (Zhang et al., 2013).

Application of exogenous SA into the nutrient solution in hydroponic systems may have substantial importance in the limitation of pathogen development (Spletzer and Enyedi, 1999; Mandal et al., 2009) and plant adaptation to excessive salinity (Tari et al., 2002). This is particularly relevant to systems with recirculation of the nutrient solution in which – as opposed to those with non-circulating systems – a major risk of increased salinity and pathogen contamination of nutrient solution occurs. This problem concerns cultivation systems with technical limitations of solution disinfection (Smoleń et al., 2015).

The efficiency of exogenous SA application in plant adaptation to stress conditions depends on the dose and time of plant treatment with SA. It has been shown that SA application increases tomato tolerance to salinity (Tari et al., 2002). In hydroponic studies conducted by Mandal et al. (2009), SA induced plant resistance to *Fusarium oxysporum* f. sp. *lycopersici*, while Spletzer and Enyedi (1999) found that SA induced plant resistance to *Alternaria solani*. Extended treatment with excessive doses of SA can, however, be harmful to plants (Jung et al., 2004).

Smoleń et al. (2015) revealed that the introduction of SA into the nutrient solution (at a dose of 1 mg SA⋅dm^-3^; i.e., 7.24 μM SA) improved the efficiency of I biofortification of tomato fruits. In addition, SA increased I accumulation in vegetative parts of tomato plants. No harmful effect of this compound on plants was observed. The improvement of I biofortification through SA application may allow for reduced cost of plant enrichment with beneficial elements (Smoleń et al., 2015).

Another important aspect is to recognize the influence of SA on the uptake and metabolism of Se in plants. It seems crucial to describe these issues with simultaneous application of I + Se and SA, as both of these elements, along with SA, are easily volatilised from plants. During the process of its methylation, CH_3_I (Itoh et al., 2009), dimethyl diselenide (DMDSe), dimethyl selenide (DMSe) (Winkel et al., 2015) and MeSA are formed (Zhang et al., 2013). Studies on the interaction among I, Se, and SA would fill the information gap with respect to the possibility of plant biofortification with these elements during the same treatment. This approach can be readily included in terms of production of the functional food. Of great importance is also the fact that the products of Se methylation may cause damage to the ozone layer. It seems advisable to determine whether exogenous SA alters the accumulation of Se in plants and to what extent it might affect molecular and biochemical mechanisms regulating the process of DMDSe or DMSe synthesis. The main difficulty in recognizing these issues is that the lettuce genome is not fully known, which hinders molecular research, including primer design for gene expression analysis.

The goal of this study was to determine the influence of exogenous SA on the process of plant biofortification simultaneously with I and Se. In addition, another goal of this study was to evaluate the application of tested compounds with respect to chemical composition and mineral nutrition of roots and leaves as well as on selenocysteine methyltransferase (*SMT*) gene expression in lettuce plants cultivated in a hydroponic NFT system.

## Materials and Methods

### Plant Material and Treatments

Cultivar ‘Zimująca’ lettuce (*Lactuca sativa* L. var. *capitata*) was cultivated during the autumn season in an NFT hydroponic system. The experiment was conducted during 2014–2015 in a greenhouse of the Faculty of Biotechnology and Horticulture, University of Agriculture in Kraków. Each year, seeds were sown into rockwool plugs (Grodan, Rockwool B.V., Roermond, Netherlands) at the turn of August and September (01.09.14 and 24.08.15, respectively). Seedlings at the two-leaf stage were placed into holes (spaced 25 cm apart) of Styrofoam slabs filling NFT beds (‘dry hydroponic’ method). No additional substrate was used. The greenhouse was equipped with five individual NFT sets with 1,300-dm^3^ medium containers, facilitating lettuce cultivation in recirculating hydroponics.

After plant seedlings were planted in the hydroponic systems, day and night temperatures were set to 15°C and 10°C, respectively. From the beginning of October to the end of the experiment, natural light was supplemented between 5.00 a.m. and 10.00 a.m. with the use of 600-W high-pressure sodium lamps.

Studies included the introduction of I (as KIO_3_ puriss. p.a., Avantor Performance Materials, Gliwice, Poland), Se (as Na_2_SeO_3_ puriss. p.a., Sigma–Aldrich, St. Louis, MO, USA) and SA (puriss. p.a., Avantor Performance Materials) into the nutrient solution in the following treatments: (1) control (trace I and Se levels in the nutrient solution from applied fertilizers of 30 μg⋅dm^-3^ I and 8.5 μg⋅dm^-3^ Se, respectively), (2) I + Se, (3) I + Se + 0.1 SA (0.1 mg SA⋅dm^-3^ nutrient solution; i.e., 0.724 μM SA), (4) I + Se + 1.0 SA (1.0 mg SA⋅dm^-3^ nutrient solution; i.e., 7.24 μM SA) and (5) I + Se + 10.0 SA (10.0 mg SA⋅dm^-3^ nutrient solution; i.e., 72.4 μM SA). KIO_3_ was used at a dose of 5 mg I⋅dm^-3^ (i.e., 39.4 μM I), while Na_2_SeO_3_ was at 0.5 mg Se⋅dm^-3^ (i.e., 6.3 μM Se). I, Se and SA were instantly introduced into the nutrient solution beginning at the 3-to-4-leaf stage (formation of the rosette). The experiment was conducted according to a randomized block design with four replications - five plants per one replicate in each treatment. Plants were grown in a nutrient solution with pH 5.50, EC 1.8 mS⋅cm^-1^ and the following contents of macro- and micronutrients (mg⋅dm^-3^): 120 N, 40 P, 170 K, 35 Mg, 150 Ca, 1.5 Fe, 0.55 Mn, 0.25 Zn, 0.2 B, 0.09 Cu and 0.04 Mo, which is equivalent to 8.57 mM N, 1.29 mM P, 4.35 mM K, 1.44 mM Mg, 3.74 mM Ca, 26.9 μM Fe, 10.0 μM Mn, 3.8 μM Zn, 18.5 μM B, 1.4 μM Cu, and 0.4 μM Mo.

For each treatment, 1,300 dm^3^ of nutrient solution were stored in separate containers and periodically administered to the cultivation slabs. The frequency of watering was adjusted for the growth stage of lettuce and weather conditions. Plants were cultivated in the recirculating system of nutrient solution without a disinfection system. Plants used the same nutrient solutions throughout the entire period.

Lettuce harvest, followed by the assessment of head and root weight and collection of leaf and root samples, was conducted on 01.12.14 and 16.12.15.

### Plant Analysis

For the analyses described in Sections “Plant Analysis of Fresh Samples” and “Plant Analysis after Sample Drying,” lettuce heads were cut in half and mixed in order to obtain a representative sample of all leaves (old and young) from all five heads in each treatment.

#### Plant Analysis of Fresh Samples

For fresh samples of lettuce leaves and roots (directly after harvest), dry matter content was assayed at 105°C. The content of L-ascorbic acid in leaves was analyzed by capillary electrophoresis after the homogenisation of 20-g samples in 80 cm^3^ of 2% oxalate acid (puriss. p.a., Avantor Performance Materials) and further centrifugation for 15 min at 4,500 rpm, 5°C. The supernatants were filtered through a 0.25-μm cellulose acetate membrane filter and analyzed using a PA 800 Plus capillary electrophoresis system (Beckman Coulter, Indianapolis, IN, USA) with diode array detector (DAD) detection. Capillaries of 50 μm i.d. and 365 μm o.d. and those of a total length of 50 cm (40 cm to detector) were used. A negative power supply of 25 kV was applied. The running buffer solution was prepared as proposed by Zhao et al. (2011), containing 30 mM NaH_2_PO_4_ (puriss. p.a., Avantor Performance Materials), 15 mM Na_2_B_4_O_7_ (puriss. p.a., Sigma–Aldrich) and 0.2 mM cetyltrimethylammonium bromide (CTAB) (puriss. p.a., Sigma–Aldrich) (pH 8.80).

In order to determine the content of sugars and phenolic compounds, fresh leaves were extracted with boiling 96% ethanol (Destylernia ‘Polmos’ Sp. z o.o., Kraków, Poland) using a reflux condenser. The levels of fructose, glucose and sucrose (and their sum as total sugars) were assessed by a PA 800 Plus capillary electrophoresis system (Beckman Coulter) with DAD detection. Capillaries of 25 μm and 365 μm o.d. and those of a total length of 60 cm (50 cm to detector) were used. A power supply of 30 kV normal polarity was applied. The detector was set at 205 nm. The running buffer solution contained 15 mL 36 mM Na_2_HPO_4_, 130 mM NaOH (puriss. p.a., Avantor Performance Materials) and 1.93 mM β-cyclodextrin (puriss. p.a., Sigma–Aldrich) (pH 12.7).

The content of phenols, phenylpropanoids, flavonols, and anthocyanins was determined spectrophotometrically after sample reactions with 0.1% HCl (puriss. p.a., Avantor Performance Materials) dissolved in ethanol (Fakumoto and Mazza, 2000).

#### Plant Analysis after Sample Drying

Fresh lettuce roots and leaves (after being washed in distilled water) were dried at 70°C in a laboratory dryer with forced air circulation and ground in a FRITSCH Pulverisette 14 variable speed rotor mill (Idar-Oberstein, Germany) using 0.5-mm sieve. Samples were subsequently analyzed with respect to the contents of I, Se, P, K, Ca, Mg, S, Na, B, Cu, Fe, Mn, Mo, and Zn by the ICP-OES technique (using an ICP-OES Prodigy Spectrometer, Leeman Labs, New Hampshire, MA, USA); N by the Kjeldahl method; and selenomethionine (SeMet; Acros Organics, Geel, Belgium), selenocysteine (SeCys; Sigma–Aldrich), proline (Sigma–Aldrich) and SA (puriss. p.a., Avantor Performance Materials) using capillary electrophoresis via a PA 800 Plus system (Beckman Coulter).

Determination of I and Se content after tetramethylammonium hydroxide (TMAH) extraction: amounts of 0.5 g air-dried leaf or root samples, 10 cm^3^ double-distilled water and 1 cm^3^ of 25% TMAH (Sigma–Aldrich) were added to 30-cm^3^ Falcon tubes. After mixing, samples were incubated for 3 h at 90°C. After incubation, samples were cooled to a temperature of approximately 20°C and filled to 30 cm^3^ with double-distilled water. After mixing, samples were centrifuged for 15 min at 4,500 rpm. The measurements of I and Se content using an ICP-OES spectrometer (Leeman Labs) were conducted in the supernatant without decanting (PN-EN 15111, 2008; Smoleń et al., 2016).

Determination of SeMet, SeCys and proline content in lettuce: in 30-cm^3^ Falcon tubes, 5 cm^3^ of solution containing 40 mg protease and 20 mg lipase in demineralised water were added to 0.1-g air-dried plant samples (leaves or roots). Samples were incubated for 16 h at 20°C and then centrifuged for 15 min at 4,500 rpm (Zhao et al., 2011). The aliquots of 0.5 cm^3^ supernatants were transferred into 1.5-cm^3^ Eppendorf tubes and centrifuged for 10 min at 10,000 rpm. Measurements of SeMet, SeCys and proline content were conducted using the capillary electrophoresis technique with laser-induced fluorescence detection (PA 800 Plus, Beckman Coulter). The derivatisation procedure by Arlt et al. (2001) was slightly modified in order to prepare extracts for the laser-induced fluorescence measurements. One hundred microliters of plant extract and 100 μL carbonate buffer + fluorescein isothiocyanate mixture (4.9 mL carbonate buffer with final pH 9.2 in the mixture + 100 μL of 210-μM fluorescein isothiocyanate dissolved in acetone), after mixing, were incubated for 2 h in the dark. An amount of 0.2 M carbonate buffer at pH 11.2 was applied (Na_2_CO_3_, trace analysis quality; Sigma–Aldrich)_._ The analogous derivatisation procedure was applied to standard reagents and blank samples (double-distilled water). SeMet, SeCys and proline were analyzed using capillaries of 50 μm i.d. and 365 μm o.d. and those of a total length of 50 cm (40 cm to the detection window). Separation was conducted for 60 min at 7 kV using the standard PA 800 Plus laser (Beckman Coulter) with 488-nm and 500-nm long-pass filters in the detector. The running buffer contained 45 mM β-cyclodextrin + 80 mM sodium borate (Sigma–Aldrich) (pH 9.2).

Determination of SA content: a plant sample amount of 0.2 g was placed in 7-cm^3^ polypropylene tubes; 5 cm^3^ of demineralised water was added and mixed for 1 min. Samples were incubated for 30 min in a 60°C water bath, cooled to room temperature and centrifuged for 15 min at 5°C at 4,500 rpm. Samples were then filtered through cellulose acetate membrane filters and analyzed by capillary electrophoresis via a PA 800 Plus system (Beckman Coulter). Capillaries of 75 μm i.d. and 365 μm o.d. and those of a total length of 30 cm (20 cm to the detector window) were used. Separation was conducted at -25 kV and the detection of SA at 205 nm. The running buffer solution contained 10 mM Tris (Sigma–Aldrich) at pH 2.78 set by formic acid (puriss. p.a., Avantor Performance Materials) (Coolen et al., 1998).

Determination of macro- and microelements: in order to analyze the total content of P, K, Ca, Mg, S, Na, B, Cu, Fe, Mn, Mo, and Zn using an ICP-OES spectrometer (Leeman Labs), plant samples were digested in 65% super pure HNO_3_ (Merck, Whitehouse, Station, NJ, USA) in a CEM MARS-5 Xpress (CEM World Headquarters, Matthews, NC, USA) microwave digestion system (Pasławski and Migaszewski, 2006). Total N content of leaf and root samples was assayed by the Kjeldahl method with the use of a VELP Scientifica UDK 193 distillation unit (Usmate, Italy).

#### Quantitative Real-Time Rerverse Transcription Polymerase Chain Reaction (qRT-PCR) Analysis of the *SMT* Gene in Leaves

Samples from the third-youngest lettuce leaf were collected directly before harvest, during the 10th week of plant cultivation in the nutrient solutions with I, Se, and SA. Three biological samples were collected simultaneously from each experimental treatment. Samples were frozen in liquid N immediately after collection and stored at -80°C until use.

The Spectrum^TM^ Plant Total RNA Kit (Sigma–Aldrich, Cat # STRN250) was used for RNA extraction of leaves, and 1 μg RNA was subsequently treated with RNase-free DNase I (Ambion Inc., Austin, TX, USA, Cat # AM2222) and 20 U⋅μL^-1^ RiboLock RNase Inhibitor (Thermo Fisher Scientific, Waltham, MA, USA, Cat # EO0381) according to the manufacturers’ instructions. RNA concentration was measured using a NanoDrop 2000c spectrophotometer (Thermo Scientific, Wilmington, DE, USA) at 260 and 280 nm, and the 260/280 nm ratio within the range of 2.00–2.15 was retained. RNA integrity and quality were verified by electrophoresis on 1% denaturing agarose gel. First-strand cDNA synthesis was performed using an iScript cDNA Synthesis Kit (Bio-Rad laboratories, Hemel Hempstead, UK, Cat #1708891) in accordance with the manufacturer’s instructions. The final concentration and quality of cDNA were determined spectrophotometrically using a Nanodrop 2000c spectrophotometer (Thermo Scientific). The 10-fold dilution products of the generated cDNAs were used for qRT-PCR analyses.

Sequences encoding the *SMT* gene, available in the GenBank database^1^, were aligned using ClustalW 2.1 software (Larkin et al., 2007). Primers were selected from conserved regions of the alignment, designated SMT-F (5′-ACACAGGAGTTGGGAATGAAG-3′) and SMT-R (5′-CTCTGATGGTGGTTGGTGTT-3′), generating a fragment of 108 bp. These sequences were submitted to BLAST of the National Center for Biotechnology Information to verify their analytical specificity, whereas the formation of dimers, hairpins and melting temperature was assessed with OligoAnalyzer 3.1 software^2^. Transcript levels were normalized to the expression level of actin (GenBank Acc. No. AB359898.1), as determined using the primers LsACT-F (5′-AGGTGTCATGGTTGGCATGGGA-3′) and LsACT-R (5′-TGTTCTTCAGGGGCGACACG-3′). The sizes of the amplified fragments were confirmed by agarose gel electrophoresis.

In addition, amplification of the serine acetyl transferase (*SAT*) gene involved in the formation of *O*-acetyl-L-serine, which represents a regulatory step in selenoamino acid assimilation, was performed (Saito, 2004). Three *SAT*-specific primer sets developed by Ramos et al. (2011) were analyzed. Unfortunately, non-specific PCR products on the agarose gels were obtained, which were inappropriate for qRT-PCR analyses.

Real-time PCR reactions consisted of 12.5 μL Maxima SYBR Green/ROX qPCR Master Mix (2x) (Thermo Fisher Scientific, Cat # K0221), 0.7 μM of each primer, 2 μL of cDNA and up to 25 μL of nuclease-free, DEPC-treated water (diethylpyrocarbonate; Thermo Fisher Scientific, Loughborough, UK, Cat # BP561-1). PCR was performed using a StepOnePlus^TM^ Real-Time PCR System (Applied Biosystems, Foster City, CA, USA) in 96-well plates with the following cycle parameters: 95°C for 10 min; 40 cycles at 95°C for 15 s; and 52°C for 1 min, and a standard melting curve (95°C for 15 s, 60°C for 1 min and 95°C for 15 s with a reading at every 0.3°C) was used to assess the specificity of PCR according to the melting temperature profile of the reference strain. Each run included a negative control and a cDNA reaction without reverse transcriptase to rule out DNA contamination. Each PCR reaction was repeated three times; therefore, the final number of repetitions for each experimental variant was three technical instrumental replicates × 2 repeated experiments × 3 biological samples.

The ΔΔC_T_ method (Livak and Schmittgen, 2001) was used to normalize and calculate ratios of expression levels (relative fold changes) relative to a housekeeping gene (actin), whose expression did not change in response to changes in the composition of the nutrition solution.

### Data Analysis

The I and Se transfer factor (TF) values in the nutrient solution-to-lettuce (leaves or roots) system were calculated using the following formula: TF = [IC or SeC_plants(dry matter)_] / (IC or SeC_nutrient solution_), where IC or SeC is the I or Se concentration, respectively.

The data were subjected to variance analysis using the analysis of variance (ANOVA) module of Statistica 10.0 PL software. It was decided to verify whether the tested factors had a significant influence on lettuce biomass as well as the analyzed parameters of the chemical composition of the plants. The Tukey test was used to determine the significance between the means at a significance level of *P* < 0.05.

#### Biofortification Target

The percentage of recommended daily allowance for I (RDA-I) and Se (RDA-Se) supplied from one serving of 50 g fresh lettuce leaves was calculated using the results of I and Se content in fresh lettuce leaves as well as the recommended daily intake of these two elements for adults: 150 μg I and 55 μg Se daily (Food and Nutrition Board – Institute of Medicine, 2000; Andersson et al., 2007).

## Results

### Lettuce Biomass

**FIGURE 1:**
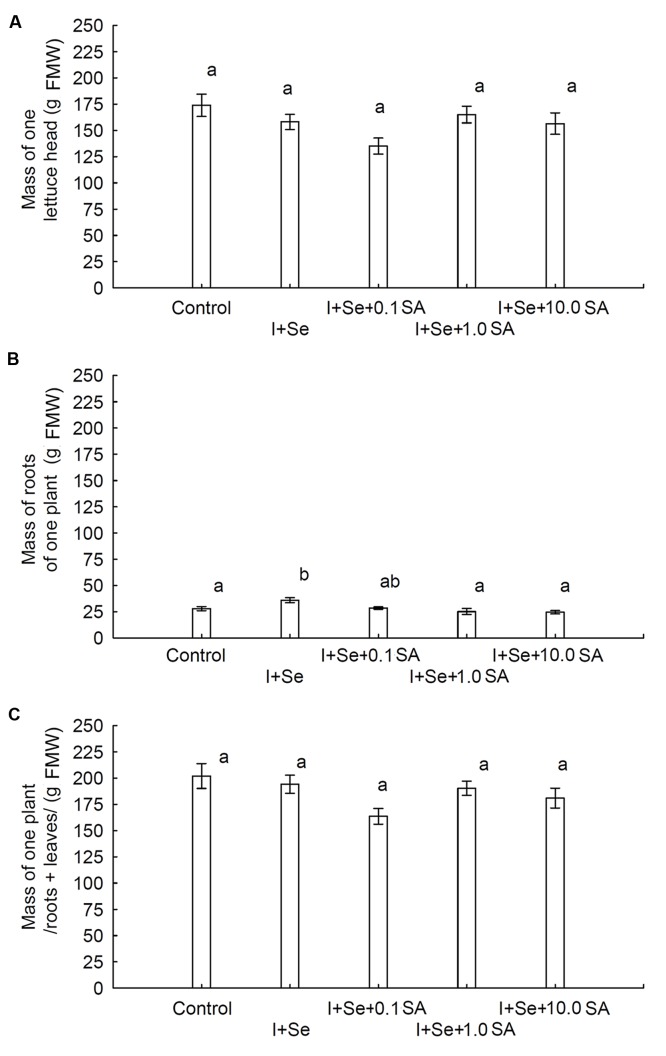
**Effects of I, Se, and salicylic acid (SA) application on the mass of a single lettuce head **(A)**, roots per plant **(B)** and whole biomass/roots + leaves per plant **(C)**.** Data are means from 2014–2015. Means followed by the same letters are not significantly different at *P* < 0.05. Bars indicate standard error (*n* = 8). FMW, fresh matter weight.

In comparison to the control, only the simultaneous application of I + Se significantly increased root biomass of lettuce. No influence of tested factors (I, Se, and SA application) on the weight of the lettuce head and total biomass (roots + leaves) was noted (**Figures 1A–C**). Root biomass was four times lower than that of lettuce leaves (heads).

### Content of I, Se and Se-Containing Amino Acids in Lettuce Plants

**FIGURE 2:**
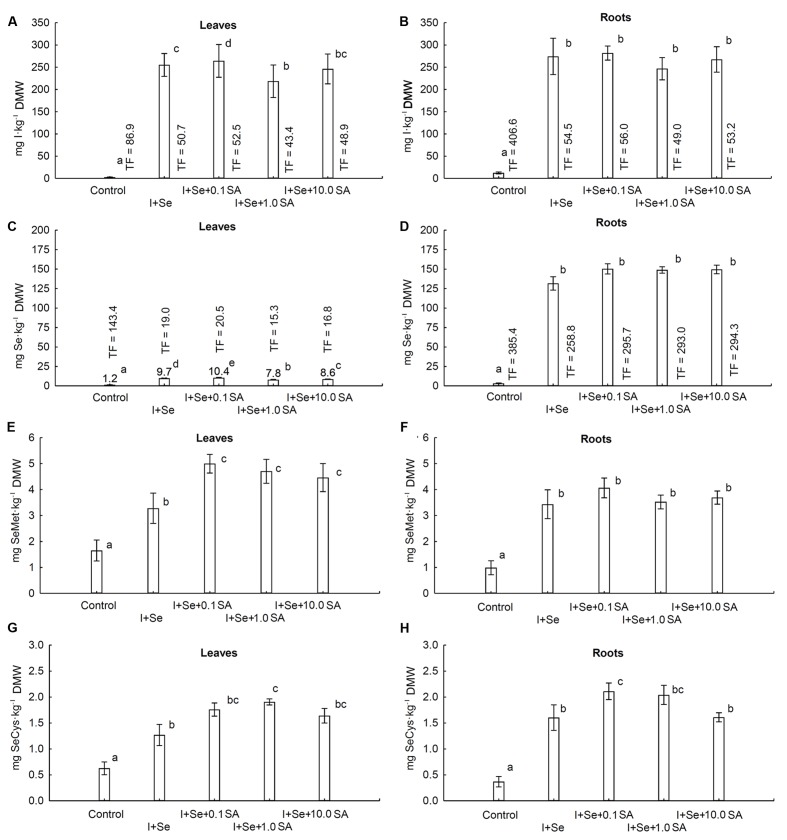
**Concentrations of I **(A,B)**, Se **(C,D)**, selenomethionine (SeMet) **(E,F)** and selenocysteine (SeCys) **(G,H)** in leaves **(A,C,E,G)** and roots **(B,D,F,H)** of lettuce.** Data are means from 2014–2015. Means followed by the same letters are not significantly different at *P* < 0.05. Bars indicate standard error (*n* = 8). DMW, dry matter weight.

A statistically significant effect of tested combinations was revealed with respect to the contents of I, Se, SeMet and SeCys in lettuce leaves and roots (**Figures 2 A–H**).

In leaves of plants grown in nutrient solutions containing I + Se separately or together with SA (at all doses), a substantial, more than 80-fold increase in I content was noted compared to the control (**Figure 2A**). The highest I accumulation in leaves was noted in plants from the I + Se + 0.1 SA combination. As compared to I + Se, additional application of 0.1 mg SA⋅dm^-3^ (I + Se + 0.1 SA) contributed to a significant increase, while addition of 1.0 mg SA⋅dm^-3^ (I + Se + 1.0 SA) contributed to a decrease of I content and TF_iodine_ values for lettuce leaves. The highest TF_iodine_ values for leaves and roots were noted in the control combination (**Figures 2A,B**). The control values of I content and TF_iodine_ for roots were 4.6 times higher than those for leaves, with I contents of roots and leaves of 12.2 and 2.6 mg I⋅kg^-1^ dry weight, respectively. In all tested combinations (I + Se with or without SA), I content in roots was more than 10 fold higher than in the control and did not significantly differ between the combinations (**Figure 2B**).

Also, with respect to Se, its content in roots of plants grown in nutrient solutions containing I + Se (separately or together with SA) was comparable between combinations and significantly higher compared to the control. Similarly, the lowest Se content in leaves was found in the control, but significant differences were noted between combinations (**Figures 2C,D**). The highest accumulation of Se in leaves was noted in plants grown with the lowest addition of SA (I + Se + 0.1 SA) in the nutrient solution. In comparison to the combination with I + Se, application of SA at the two highest doses (I + Se + 1.0 SA and I + Se + 10.0 SA) significantly decreased the Se content in leaves to an extent of 1.0 mg SA⋅dm^-3^. On average, in all tested combinations, Se content and TF_selenium_ values of roots were approximately 16 times higher than those of leaves. For the control, the value of TF_selenium_ for roots was 2.7 times higher than that of leaves.

The contents of SeMet and SeCys in leaves and roots remained comparable in all tested combinations and were significantly higher than those in the control (**Figures 2E–H**). In comparison to the application of I + Se alone, exogenous introduction of SA (at each dose) increased the levels of SeMet and SeCys in leaves (**Figures 2E,G**) and of SeCys in roots to a similar extent, with the exception of the combination of I + Se + 10.0 SA (**Figure 2H**). No significant effect of any SA dose was, however, revealed with respect to SeMet accumulation in roots (**Figure 2F**).

### Content of Proline in Lettuce Plants

**FIGURE 3:**
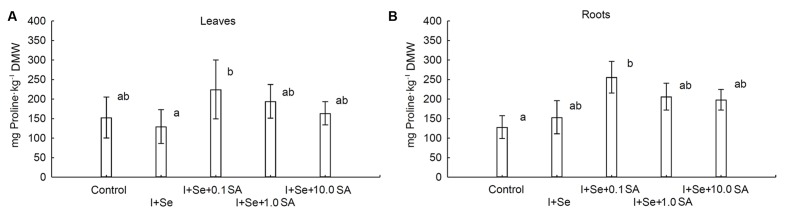
**Proline concentrations in leaves **(A)** and roots **(B)** of lettuce.** Data are means from 2014–2015. Means followed by the same letters are not significantly different at *P* < 0.05. Bars indicate standard error (*n* = 8). DMW, dry matter weight.

Introduction of I, Se and SA into the nutrient solution significantly affected proline accumulation in lettuce leaves and roots (**Figures 3A,B**). In comparison to the control, significant increases in proline levels in leaves and roots were noted in plants from the I + Se + 0.1 SA combination. The lowest level of this amino acid was noted in leaves of lettuce cultivated in the presence of I + Se in the nutrient solution. Proline concentrations in leaves and roots were of similar magnitude.

### Content of SA in Lettuce Plants

**FIGURE 4:**
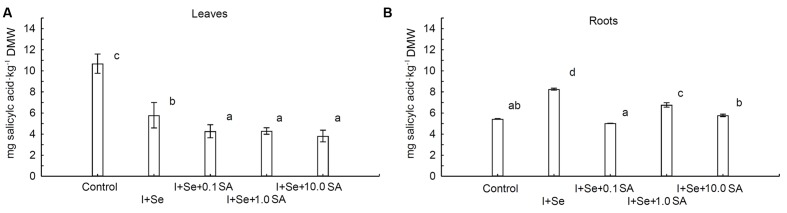
**Effects of I, Se and salicylic acid (SA) application on the concentrations of SA in leaves **(A)** and roots **(B)** of lettuce.** Data are means from 2014–2015. Means followed by the same letters are not significantly different at *P* < 0.05. Bars indicate standard error (*n* = 8). DMW, dry matter weight.

The contents of SA in leaves and roots were strongly dependent upon the introduction of I, Se, and SA into the nutrient solution (**Figures 4A,B**). Compared to the control, a diverse influence of applied compounds on the SA level was noted for leaves and roots. In the case of leaves, all tested compounds significantly decreased the SA content compared to the control (**Figure 4A**). The lowest but comparable level of SA was assayed in leaves of plants grown in the presence of SA in the nutrient solution (0.1, 1.0 and 10.0 mg SA⋅dm^-3^). Plants treated with I + Se were characterized by leaf levels of SA between those of the control combination and those of SA introduction.

Conversely, in roots of plants cultivated in the nutrient solution enriched with I + Se, I + Se + 1.0 SA and I + Se + 10.0 SA, the content of SA was significantly higher compared to the control. The highest concentration of SA was noted in the I + Se combination. Simultaneous application of I + SA + 0.1 SA had no effect on SA levels in lettuce roots.

### *SMT* Gene Expression in Leaves

**FIGURE 5:**
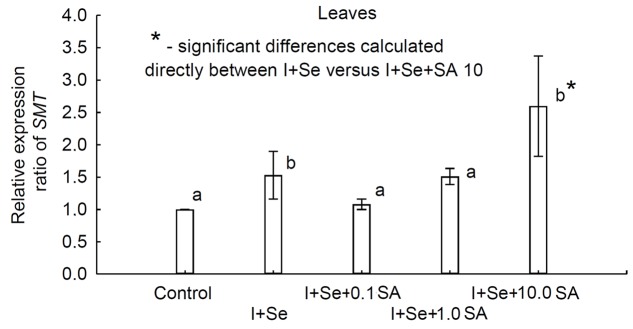
**Relative expression ratio based on quantitative real-time reverse transcription polymerase chain reaction (qRT-PCR) analysis of the selenocysteine methyltransferase (*SMT*) gene in lettuce leaves.** Relative *SMT* gene expression levels in samples from all treatments were expressed as fold changes in comparison to the control using the method of Livak and Schmittgen (2001). Actin was used to normalize input amounts of RNA and the level of target gene expression. Values represent the means ± standard error for *n* = 18, and the asterisk indicates statistical significance (*P* < 0.05).

qRT-PCR was used to quantify the expression levels of the *SMT* gene in leaves of lettuce grown in different nutrient solutions. The results showed, compared to the control, that the *SMT* gene appeared to be up-regulated (**Figure 5**). Differential expression patterns for *SMT* were found across the individual treatments. A significantly greater expression level (compared to the control) was determined for plants grown in nutrient solutions with I + Se and I + Se + 10.0 SA. Moreover, the relative expression pattern was significantly higher for plants grown on I + Se + 10.0 SA media compared to those cultivated on media without SA (I + Se). No significant differences in transcript abundance were detected for plants treated with I + Se + 0.1 SA and I + Se + 1.0 SA and also in comparison with plants cultivated in nutrient solutions with the addition of I + Se.

### Chemical Composition of Lettuce Plants

#### Dry Matter Content and Nutritional Quality of Lettuce

Tested factors had a significant influence on the content of sugars (fructose, glucose, sucrose, and their sum) but did not affect the dry matter content (leaves and roots) and the content of ascorbic acid, phenolic compounds, phenylpropanoids, flavonols and anthocyanins in lettuce leaves (**Tables 1** and **2**).

**Table 1:** Concentration of ascorbic acid in leaves and dry weights of leaves and roots of lettuce.

Treatment	Ascorbic acid (mg⋅100g^-1^ FMW)	Percentage of leaf DMW	Percentage of root DMW
Control	5.2 ± 1.5a	4.76 ± 0.17a	5.43 ± 0.34a
I + Se	6.8 ± 1.6a	5.22 ± 0.21a	5.42 ± 0.26a
I + Se + 0.1 SA	6.7 ± 1.9a	5.28 ± 0.19a	5.56 ± 0.23a
I + Se + 1.0 SA	6.6 ± 1.5a	4.93 ± 0.24a	4.84 ± 0.22a
I + Se + 10.0 SA	4.8 ± 1.3a	5.10 ± 0.17a	5.04 ± 0.29a


*Data are means from 2014–2015 (*n* = 8). Means followed by the same letters are not significantly different at *P* < 0.05. FMW, fresh matter weight; DMW, dry matter weight.*

**Table 2:** Concentration of fructose, glucose, sucrose, total sugars, phenolic compounds, phenylpropanoids, flavonols, and anthocyanins in lettuce leaves.

Treatment	(mg⋅100 g^-1^ FMW)			
	
	Fructose	Glucose	Sucrose	Total sugars
Control	120.7 ± 24.8a	83.2 ± 18.3a	95.2 ± 11.2a	299.1 ± 48.9a
I + Se	213.8 ± 21.0bc	156.0 ± 18.8b	139.8 ± 13.0b	509.5 ± 45.3b
I + Se + 0.1 SA	166.3 ± 22.1ab	128.0 ± 18.0ab	146.9 ± 14.0b	441.2 ± 41.2b
I + Se + 1.0 SA	223.9 ± 27.9c	152.5 ± 19.4b	129.5 ± 13.6ab	505.8 ± 43.1b
I + Se + 10.0 SA	234.3 ± 11.7c	167.2 ± 11.3b	125.3 ± 13.2ab	526.8 ± 22.1b

	**(mg⋅100 g^-1^ FMW)**			
	
	**Phenolic compounds**	**Phenylpropanoids**	**Flavonols**	**Anthocyanins**

Control	187.0 ± 19.2a	45.1 ± 2.8a	62.1 ± 4.0a	29.9 ± 2.7a
I + Se	205.2 ± 11.4a	50.1 ± 3.2a	68.8 ± 4.6a	33.7 ± 3.2a
I + Se + 0.1 SA	209.7 ± 17.8a	51.3 ± 2.2a	70.6 ± 3.1a	34.6 ± 1.7a
I + Se + 1.0 SA	198.5 ± 12.2a	48.5 ± 3.3a	66.8 ± 4.9a	32.9 ± 2.9a
I + Se + 10.0 SA	204.2 ± 12.6a	50.0 ± 3.4a	68.2 ± 4.5a	32.7 ± 2.3a


*Data are means from 2014–2015 (*n* = 8). Means followed by the same letters are not significantly different at *P* < 0.05. FMW, fresh matter weight.*

In comparison to the control, all tested combinations with I + Se applied separately or together with SA contributed to a comparable increase in total content of sugars in leaves (**Table 1**). In comparison to the control, significant and comparable increases of fructose and glucose were noted in the combinations with I + Se, I + Se + 1.0 SA and I + Se + 10.0 SA. Only the introduction of I + Se or I + Se + 0.1 SA into the nutrient solution increased sucrose accumulation in leaves relative to the control. No tested combination decreased the level of sugars in lettuce plants.

#### Nutritional Status of Lettuce Plants

A significant and diverse influence of tested factors on the content of macro- and micronutrients in lettuce was noted (**Tables 3** and **4**). The only exception was the lack of significant differences in Ca and Mo accumulation in lettuce among combinations.

**Table 3:** Concentrations of N, P, K, Ca, Mg, S, and Na in leaves and roots of lettuce.

Part Of plant	Treatment	Percentage of DMW					
		
		N	P		K		Ca
Leaves	Control	5.06 ± 0.07b	0.69 ± 0.01b		8.34 ± 0.16b		1.10 ±0.06a
	I + Se	4.95 ± 0.11ab	0.69 ± 0.02b		7.60 ± 0.21a		0.95 ± 0.06a
	I + Se + 0.1 SA	5.02 ± 0.03ab	0.65 ± 0.01ab		7.94 ± 0.20ab		1.05 ± 0.07a
	I + Se + 1.0 SA	4.78 ± 0.05a	0.63 ± 0.02a		7.94 ± 0.13ab		0.98 ± 0.09a
	I + Se + 10.0 SA	4.91 ± 0.04ab	0.62 ± 0.02a		8.08 ± 0.14ab		1.05 ± 0.04a
Roots	Control	3.09 ± 0.02a	1.09 ± 0.20a		4.45 ± 0.03a		5.30 ± 0.07d
	I + Se	3.28 ± 0.05ab	1.45 ± 0.07c		4.46 ± 0.09a		4.47 ± 0.05c
	I + Se + 0.1 SA	3.36 ± 0.10bc	1.73 ± 0.06d		5.32 ± 0.23d		4.18 ± 0.15b
	I + Se + 1.0 SA	3.55 ± 0.07c	1.45 ± 0.16c		5.11 ± 0.05c		3.52 ± 0.12a
	I + Se + 10.0 SA	3.20 ± 0.06ab	1.33 ± 0.16b		4.61 ± 0.08b		4.12 ± 0.04b

		**Mg**		**S**		**Na**	

Leaves	Control	0.34 ± 0.01c		0.17 ± 0.005b		0.11 ± 0.004a	
	I + Se	0.32 ± 0.01bc		0.15 ± 0.005a		0.14 ± 0.006b	
	I + Se + 0.1 SA	0.24 ± 0.01a		0.15 ± 0.004a		0.14 ± 0.007b	
	I + Se + 1.0 SA	0.30 ± 0.02b		0.16 ± 0.008ab		0.13 ± 0.009b	
	I + Se + 10.0 SA	0.32 ± 0.01bc		0.16 ± 0.006ab		0.14 ± 0.003b	
Roots	Control	1.13 ± 0.01d		0.26 ± 0.004a		0.41 ± 0.006e	
	I + Se	0.78 ± 0.03c		0.25 ± 0.007a		0.27 ± 0.014d	
	I + Se + 0.1 SA	0.58 ± 0.04a		0.32 ± 0.007c		0.19 ± 0.017a	
	I + Se + 1.0 SA	0.58 ± 0.02a		0.30 ± 0.007b		0.21 ± 0.003b	
	I + Se + 10.0 SA	0.73 ± 0.02b		0.25 ± 0.008a		0.23 ± 0.009c	


*Data are means from 2014–2015 (*n* = 8). Means followed by the same letters are not significantly different at *P* < 0.05. DMW, dry matter weight.*

**Table 4:** Concentrations of B, Cu, Fe, Mn, Mo, and Zn in leaves and roots of lettuce.

Part of plant	Treatment	mg⋅kg^-1^ DMW		
		
		B	Cu	Fe
Leaves	Control	32.4 ± 2.2b	9.2 ± 0.7b	152.9 ±19.1bc
	I + Se	29.3 ± 1.6a	6.6 ± 0.1a	124.8 ± 13.7a
	I + Se + 0.1SA	31.9 ± 2.5ab	13.4 ± 2.8c	181.4 ± 16.9d
	I + Se + 1.0 SA	30.9 ± 1.9ab	5.9 ± 0.2a	134.4 ± 18.6ab
	I + Se + 10.0 SA	30.8 ± 1.4ab	7.3 ± 0.8ab	154.8 ± 15.0c
Roots	Control	30.4 ± 3.80d	134.5 ± 8.5e	14,548.1 ± 1,331.4d
	I + Se	25.9 ± 2.18b	84.1 ± 8.5d	6,274.0 ± 1,550.3c
	I + Se + 0.1 SA	22.5 ± 1.79a	71.1 ± 9.6b	6,570.4 ± 1,489.6c
	I + Se + 1.0 SA	25.7 ± 1.63b	55.3 ± 0.4a	3,517.7 ± 1,348.6a
	I + Se + 10.0 SA	27.0 ± 2.60c	76.3 ± 2.2c	4,739.5 ± 1,109.2b

		**mg⋅kg^-1^ DMW**		
		
		**Mn**	**Mo**	**Zn**

Leaves	Control	67.4 ± 6.4a	1.5 ± 0.2a	34.9 ± 2.5c
	I + Se	62.2 ± 5.1a	1.2 ± 0.1a	22.9 ± 1.0a
	I + Se + 0.1 SA	109.6 ± 14.1b	1.5 ± 0.1a	29.2 ± 0.9b
	I + Se + 1.0 SA	83.3 ± 8.3a	1.3 ± 0.2a	21.7 ± 0.6a
	I + Se + 10.0 SA	61.2 ± 10.8a	1.3 ± 0.1a	22.6 ± 0.9a
Roots	Control	980.0 ± 20.4d	23.4 ± 4.6c	119.8 ± 9.6c
	I + Se	709.5 ± 54.9b	19.4 ± 2.3b	113.8 ± 4.9c
	I + Se + 0.1 SA	799.1 ± 90.4c	14.1 ± 0.9a	117.4 ± 4.3c
	I + Se + 1.0 SA	560.1 ± 20.4a	19.1 ± 2.3b	98.8 ± 5.5b
	I + Se + 10.0 SA	814.1 ± 22.8c	16.0 ± 1.2a	86.0 ± 4.8a


*Data are means from 2014–2015 (*n* = 8). Means followed by the same letters are not significantly different at *P* < 0.05. DMW, dry matter weight.*

In roots of plants from all combinations, a significant increase of P concentration was found with respect to the control. In addition, there was an increase of N and S content after the application of Se + 0.1 SA and I + Se + 1.0 SA as well as of K in the combinations with I + Se + SA at all SA doses. A negative effect of I + Se and I + Se + SA was mainly manifested by the decreased contents of Ca, Mg, Na (**Table 3**), B, Cu, Fe, Mn, Mo, and Zn (**Table 4**). In each case, lower levels of these nutrients in roots were revealed compared to the control. To the greatest extent, a decrease of the content of analyzed nutrients in lettuce roots was noted in the respective combinations: Ca, Cu, Fe, and Mn after the application of I + Se + 1.0 SA; Na after the application of I + Se + 0.1 SA; Zn after the application of I + Se + 10.0 SA; Mg comparably in the combinations with I + Se + 0.1 SA and I + Se + 1.0 SA; and Mo comparably in the combinations with I + Se + 0.1 SA and I + Se + 10.0 SA.

Introduction into the nutrient solution of I, Se, and SA improved the accumulation of Na in lettuce leaves compared to the control, but the increase did not vary between tested combinations (**Table 3**). In addition, the application of I + Se + 0.1 SA led to the increase of Cu, Fe, and Mn content in leaves (**Table 4**). In all remaining combinations, a decrease of Cu content in lettuce was noted, with no statistical significance for I + Se + 10.0 SA. In the case of Fe, a decrease in its concentration was found in leaves of plants grown in the presence of I + Se and I + Se + 1.0 SA. A tendency of diminishing N, P, K, Mg, S, B, and Zn contents in lettuce leaves was noted for all tested combinations compared to the control (**Tables 3** and **4**), but statistically significant changes were noted only for K, S, B, Cu, Fe, and Zn after the application of I + Se.

It should be noted that, compared to the control, all tested treatments led to simultaneous decreases in Mg contents in leaves and roots. In the case of Zn, only for the applications of I + Se + 1.0 SA and I + Se + 10.0 SA did its contents decrease in both above- and belowground parts of plants.

#### Biofortification Target

The increase in the content of I and Se in lettuce after the application of these two elements allowed for increasing the possibility of covering RDAs for both nutrients after the consumption of a single 50-g serving of lettuce leaves (**Table 5**). In comparison to the control, RDA-I values increased from 3.5% to 388.8–441.1% for I + Se + 0.1 SA and I + Se + 10.0 SA, respectively. In the case of RDA-Se, growth was less distinctive (i.e., from 5.6% to 39.1–53.7% for I + Se + 1.0 SA and I + Se, respectively).

**Table 5:** Value of the I:Se molar mass ratio and percentage of recommended daily allowance (RDA) for I and Se in a 50-g portion of fresh lettuce leaves.

Treatment	Percentage RDA for I	Percentage RDA for Se	I:Se molar mass ratio
Control	3.5 ± 1.0a	5.6 ± 1.8a	1.3:1 ± 0.2:1a
I + Se	427.6 ± 56.1c	53.7 ± 4.0e	13.1:1 ± 0.9:1b
I + Se + 0.1 SA	388.8 ± 76.8b	50.2 ± 3.0d	12.4:1 ± 1.9:1b
I + Se + 1.0 SA	422.6 ± 84.9c	39.1 ± 3.4b	17.0:1 ± 2.2:1d
I + Se + 10.0 SA	441.1 ± 81.7c	47.8 ± 4.4c	14.7:1 ± 1.6:1c


*Data are means from 2014–2015 (*n* = 8). Means followed by the same letters are not significantly different at *P* < 0.05.*

Introduction of I, Se and SA into the nutrient solution widened the molar ratio of I and Se content in lettuce plants from 1.3:1 (control) to 12.4:1 (I + Se + 0.1 SA) and 17.0:1 (I + Se + 1.0 SA).

## Discussion

### Plant Biomass and Chemical Composition of Lettuce Plants

The results obtained in this study indicate that SA application (at three different doses), when combined with I + Se, has no adverse influence on lettuce growth and development and, consequently, on leaf and root biomass. Tested compounds also did not affect dry matter content and the synthesis of ascorbic acid, sugars and secondary metabolites from the group of phenolic compounds, phenylpropanoids, flavonols and anthocyanins in lettuce plants. Smoleń et al. (2015) revealed that simultaneous application of KIO_3_ + SA (1 mg SA⋅dm^-3^ (i.e., 7.24 μM SA)) through the nutrient solution, compared to the application of KIO_3_ alone, significantly increased the accumulation of nitrates(V) and decreased total acidity and the soluble solid (% Brix), fructose and glucose contents in fruits of tomato plants grown in an NFT system. Such dependencies were not, however, noted for simultaneous application of KI and SA compared to treatment only with KI. The authors revealed that KI + SA application also reduced the ascorbic acid content of tomato fruits.

Results of the present study, compared to those obtained by Smoleń et al. (2015), show that the effects of SA introduction into the nutrient solution depend strongly on the crop species and the analyzed part of the plant – in this case, lettuce leaves or tomato fruits.

It is worth mentioning that in hydroponic cultivation, even small doses of I (Blasco et al., 2010; Kato et al., 2013) and Se (Ríos et al., 2008, 2010; Hawrylak-Nowak et al., 2015) can be harmful to plants. Also, SA, when applied for a long time at low concentrations or for a short period at high doses, can cause plant damage (Jung et al., 2004).

No negative impacts of tested combinations noted in our study on plant biomass confirmed the application of safe doses of the compounds. No visual symptoms of plant damage due to toxicity were observed.

#### Mineral Nutrition; Efficiency of I and Se Biofortification; and *SMT* Gene Expression

Leafy vegetables belong to a group of plants that accumulate substantial amounts of I (Dai et al., 2004; Hong et al., 2008). On the other hand, vegetables from this group (spinach, pak choi) exhibit higher sensitivity to I fertilization than, for example, carrot (Dai et al., 2004).

Some plant species, including those of the *Asteraceae* family with lettuce, are described as hyperaccumulators. They can accumulate Se up to 100 times more compared to other vegetable crops (Beath et al., 1939a,b). In hyperaccumulators, Se is mainly present in the methylated form (methyl-SeCys) but not SeMet and SeCys. The accumulation of SeMet and SeCys occurs in non-accumulators – those plants that take up small amounts of this element (Yuan et al., 2013). The process of SeCys transformation into methyl-SeCys (by the SMT enzyme) in the Se metabolism pathway plays a protective role for plants. It consists of the conversion of potentially toxic Se-amino acids into less harmful selenometabolites (Ellis et al., 2004).

According to Winkel et al. (2015), the process of SeCys conversion to methyl-SeCys is one of the four independent metabolic pathways responsible for the synthesis of various selenoorganic compounds from SeCys. With the participation of L-cysteine oxidase, methyl-SeCys undergoes transformation into methylselenocysteine selenoxide (MSeCysSeO), which is further converted by cysteine sulphoxide lyase to volatile DMDSe. DMDSe also can be formed in another pathway converting SeMet into Se-methylseleno-L-cysteine. Se-Met alone is synthesized through conversion of SeCys by a few steps.

Another volatile Se compound that is formed through methylation and undergoes volatilisation is DMSe, produced in a separate few-step metabolic pathway from SeMet. Volatilisation of DMDSe and DMSe contributes to a decrease of Se content in plant leaves (Winkel et al., 2015).

In the combination with I + Se application (compared to the control), *SMT* gene expression in lettuce substantially increased, which seems justified by the content of total Se and SeMet. Interpretation of the results is, however, far more complex (**Table 6**). Aside from the analyzed *SMT* gene expression, chemical analyses of leaves and roots clearly indicate that, depending on the dose, exogenous SA (applied together with I + Se) has a significant and diverse effect on plants, mostly with respect to the syntheses of SeMet, SeCys and proline in leaves and roots. In addition, significant differences were observed in SA accumulation and the functioning of mineral nutrition, including the uptake and accumulations of I and Se as well as macro- and micronutrients in lettuce leaves and roots.

**Table 6:** Overview of the maximum and minimum results (from **Tables 1**–**5** and **Figures 1–5**) obtained for leaves and roots of lettuce grown in hydroponic solution supplemented with I, Se and different doses of SA.

Treatment	Leaves		Roots	
		
	*In* +	*In* -	*In* +	*In* -
Control	—	—	—	SA
I + Se	Sucrose	K, S, B, Cu, Fe, Zn	Mass of roots	—
I + Se + 0.1 SA	I	SA	Proline	SA
	Se	Mg, S	N, P, K, S	SeCys
	SeMet			Mg, Na, B, Mo
	I:Se molar mass ratio			
	Se RDA			
	Glucose,			
	Sucrose			
	Proline			
	Cu, Fe, Mn			
I + Se + 1.0 SA	SeCys	I	SeCys	Ca, Mg, Cu, Fe, Mn
	Glucose	SA	N	
	Fructose	N, P, Cu, Zn		
I + Se + 10.0 SA	I-RDA	SA	—	SeCys
	*SMT* gene activity	P, Zn		Mo, Zn	
	Glucose			
	Fructose			


*‘*In +*’ *-* means ‘the highest/best’ (the extreme *In*+) values of individual character parameters from the data presented in **Tables 1**–**5** and **Figures 1**–**5**, respectively. ‘*In -*’ *-* means ‘the lowest/worst’ (the extreme *In* -) values of individual character parameters from the data presented in **Tables 1**–**5** and **Figures 1**–**5**, respectively.*

It needs to be mentioned that I also undergoes methylation in plants, forming volatile CH_3_I, a process regulated by *S*-adenosylmethionine-dependent halide/thiol methyltransferase (Itoh et al., 2009). Aside from this, plants have the ability to conduct SA methylation into MeSA via SA carboxyl methyltransferase (Tieman et al., 2010). All of these processes (syntheses of DMDSe, DMSe, CH_3_I and MeSA) require an energy input. In the available literature, there is no information on whether a common mechanism regulating these processes occurs or whether they act independently.

In studies conducted by Smoleń et al. (2014), the application of I and/or Se (alone or simultaneously) to the nutrient solution had no effect on the contents of N, P, K, Ca, Mg, S, B, Cu, Fe, Mn, Mo, and Zn in leaves and roots of lettuce cultivated in an NFT system. Importantly, introduction into the nutrient solution of KIO_3_ + Na_2_SeO_4_ (1 mg I + 0.5 mg Se⋅dm^-3^ and 1 mg I + 1.5 mg Se⋅dm^-3^) did not affect the contents of macro- and micronutrients in lettuce leaves and roots compared to the control. The causes of obtaining adverse results compared to those presented by Smoleń et al. (2014) might include the use of another lettuce cultivar and application of the chemical form of Se – Na_2_SeO_3_ instead of Na_2_SeO_4_ (Smoleń et al., 2014). In both studies, I was applied in the iodate form (KIO_3_). When plants were cultivated in hydroponics, diverse actions of Se(IV) and Se(VI) on lettuce (Ríos et al., 2008, 2010) and cucumber were noted (Hawrylak-Nowak et al., 2015). Blasco et al. (2012) revealed a diverse influence of I^-^ and ${IO}_{3}^{-}$ introduced through the nutrient solution on the mineral composition of lettuce plants. This effect was strongly dependent on the dose, including a substantial decrease of N, P, and K content in lettuce below the optimal range for this species after the application of 80 μM I^-^. Conversely, introduction of ${IO}_{3}^{-}$ affected the mineral nutrition of lettuce plants to a lesser extent, even improving the levels of Mg and Fe in plants. It needs to be mentioned that in the studies by Smoleń et al. (2014), simultaneous application of KIO_3_ + Na_2_SeO_4_ had no effect on the uptake and accumulation of I and Se (in roots and leaves) compared to plants grown in the nutrient solutions containing I or Se separately.

Blasco et al. (2012) presented results concerning macro- and micronutrient sufficiency intervals in mature leaves of lettuce plants. A thorough analysis of these data indicates that the content of macro- and micronutrients in plants from the present study stayed within the optimal range for lettuce. In the case of K, its content in leaves was even supra-optimal. This could indicate that tested doses of I, Se, and SA did not exceed the tolerance level for lettuce plants (no decrease of plant biomass) while substantially modifying the mineral nutrition (in both leaves and roots).

Smoleń et al. (2015) revealed that introduction into the nutrient solution of KI + SA and KIO_3_ + SA significantly improved I accumulation in tomato plants (in leaves and fruits) compared to plants grown in nutrient solution containing only KI or KIO_3_. Importantly, the increase of I content in tomato leaves and fruits after the application of KIO_3_ + SA was a few dozen times higher than that after the application of KI + SA. Smoleń et al. (2014, 2015) did not document molecular or biochemical mechanisms regulating the uptake and accumulation of I, Se or macro- and micronutrients and, in the case of tomato, also after the exogenous application of SA (Smoleń et al., 2015).

In our opinion, results of the present study do not provide a clear interaction at the biochemical and molecular levels among the metabolic pathways of I, Se, and SA. The results can only describe to what extent additional application of SA modifies the effect of simultaneous application of I + Se on plants.

Proline is an amino acid synthesized by plants in response to biotic or abiotic stresses (Hayat et al., 2012). In turn, SA stimulates plant reaction/resistance mechanisms to both types of stress factors (Hayat et al., 2012). Despite the lack of visual symptoms of toxic effects of tested compounds on plants, the highest concentration of proline was noted in leaves and roots of lettuce grown in the nutrient solution containing I + Se + 0.1 SA. This might have resulted from the greatest accumulation of I, Se, SeMet, Cu, Fe, and Mn in leaves and SeCys in roots of these plants. A lower expression of the *SMT* gene also was observed in plants of that treatment compared to plants that received the application of I + Se. Therefore, SA application at the lowest dose (0.1 mg SA⋅dm^-3^) may have limited Se methylation and volatilisation in the form of DMDSe by SMT enzymatic activity. The whole assessment of the conducted molecular and chemical analyses does not provide justification for the hypothesis that the dose of 0.1 mg SA⋅dm^-3^ was a stress factor to plants. This approach is further confirmed by the lack of differentiation in the content of phenolic compounds, the synthesis of which is also triggered by stresses (Ríos et al., 2008). Two hypotheses can be stated that explain the obtained results. The first one indicates that the decrease in *SMT* gene expression (combination of I + Se + 0.1 SA versus I + Se) could have resulted indirectly from the stimulation, through 0.1 mg SA⋅dm^-3^ application, of more intensive catabolism (Zhang et al., 2013) or from SA methylation processes (Tieman et al., 2010). The other hypothesis includes the limitation of *de novo* synthesis of endogenous SA (Hayat et al., 2010) in lettuce leaves and roots after the application of 0.1 mg SA⋅dm^-3^. In our opinion, introduction of exogenous SA, even at such a low dose, could have been sufficient to initiate the processes suggested in both hypotheses. As an effect, SA applied at the doses of 0.1 and 1.0 mg⋅dm^-3^ could have been a key factor limiting the expression of *SMT* (I + Se versus I + Se + 0.1 SA as well as I + Se versus I + Se + 1.0 SA).

In this aspect, surprising was a significant increase in *SMT* expression (the highest relative expression ratio value) after application of the highest dose of SA (I + Se + 10.0 SA). In this combination, the tendency of decreasing (compared to I + Se + 0.1 SA) total contents of Se in leaves and SeCys in roots indicates that a 10.0-mg SA⋅dm^-3^ dose of SA could have improved the methylation and volatilisation of DMDSe and/or DMSe. Lack of a negative influence of simultaneous application of I + Se + 10.0 SA on the sugar content in leaves indirectly suggests that photosynthesis was unaffected by tested factors and that it provided sufficient amounts of metabolites to cover the energy requirements for the mentioned processes.

Selenocysteine methyltransferase plays an important role in plant Se metabolism (Malagoli et al., 2015). Lyi et al. (2007) showed that *SMT* expression was dramatically up-regulated in broccoli plants exposed to selenate but was low in plants supplied with selenite. Furthermore, it needs to be mentioned that diverse plant species may exhibit different mechanisms of regulating *SMT* gene expression after the application of Se(IV) (i.e., ${SeO}_{3}^{2^{-}}$ ion). In Se(IV)-tolerant mutants of *Arabidopsis thaliana*, after application of exogenous ${SeO}_{3}^{2^{-}}$ , expression of the *SMT* gene is up-regulated. In plants sensitive to Se(IV), ${SeO}_{3}^{2^{-}}$ decreases the expression of that gene (Zhang et al., 2006). The results of our study indirectly indicate that expression of the *SMT* gene in lettuce additionally can be regulated by exo-/endogenous SA or by its metabolic processes in plants.

Molecular aspects of Se, I and SA metabolism should become the goal of future studies to point out the enzyme-encoding genes, whose expression is modified by the tested factors, leading to increased accumulation of certain metabolites, whose synthesis is regulated by these enzymes.

Studies concerning lettuce biofortification with Se and I should be directed to integrate analyses at the genomic, transcriptomic, proteomic and metabolomic levels. Multi-omic approaches would allow for the characterisation of individual metabolic pathways and their interrelationships. A substantial difficulty in the identification and analysis of genes related to I and Se metabolism is, however, due to the fact that the lettuce genome is not fully known.

#### Biofortification Target

The optimal molar ratio of I:Se in daily food intake stays within 4.4–8.8:1, depending on age and gender. The RDAs for these elements are 150 μg I and 55 μg Se for adults and 200–300 μg I and 60–70 μg Se for pregnant and lactating women (Food and Nutrition Board – Institute of Medicine, 2000; Andersson et al., 2007).

A substantial but supra-optimal effect of lettuce biofortification with I and Se was obtained with respect to the possibility of balancing the diet concerning I and Se RDAs. In the case of I, the consumption of 50 g fresh leaves of lettuce would supply approximately four times more I than required. In the case of Se, 50 g lettuce leaves would supply 40–50% of recommended Se intake. In our consideration, the level of lettuce enrichment of both elements can be considered too high, as it is required to balance the entire daily intake with respect to the content of I and Se.

Regarding mild deficiencies of I and Se in the population, biofortification of lettuce or other leafy vegetables (and also other crop species) with I and Se should be conducted to cover the RDA percentage for I and Se (supplied with one serving) to approximately 10–30%. This results from the need to fully balance the diet with I and Se. For severe deficiencies of both elements, the applied doses of I and Se need to be higher, so the biofortified plants should become a substantial source of these micronutrients. It seems crucial to aim to achieve the optimal molar I:Se ratio in biofortified plants. With respect to the biofortification effect, it should not contribute to a decrease in the content of other mineral nutrients in plants, particularly Fe and Zn, whose deficiencies are a widespread problem throughout the world (White and Broadley, 2009). In our studies, plant biofortification with I and Se (without SA application) negatively affected the content of K, S, B, Cu, Fe, and Zn in lettuce plants, which would result in their lower introduction to the human diet.

## Conclusion

Both tested elements are not essential to plants. These elements are, however, included in the group of elements beneficial to plants. There is a wide deficiency of I and Se in human (and other animal) diets around the world. Simultaneous plant biofortification of I and Se is necessary due to the important roles these elements play in thyroid function.

In comparison to the control, simultaneous application of both elements, aside from their improved uptake, increased the content of sugars in lettuce. Some fluctuations in the functioning of mineral nutrition in plants after the application of relatively high doses of I and Se were noted. They did not, however, affect biomass productivity of plants.

Salicylic acid, depending on the dose, modified the effects of I and Se on plants. It was revealed that when applied at a dose of 0.1 mg SA⋅dm^-3^, SA improved the efficiency of lettuce enrichment in I and Se to the greatest extent. In the case of Se, this was related to a decrease of *SMT* expression, which could have limited Se volatilisation through methylation. Limitation of Se methylation may have a positive impact on the environment, as volatile Se compounds (DMDSe or DMSe) can damage the ozone layer.

The obtained results indicate that the introduction of SA to the nutrient solutions in hydroponic systems may allow for reducing the cost of I and Se biofortification of crop plants by application of lower doses of both beneficial elements. Regarding their joint introduction with SA, it seems possible to increase their accumulation in plants to a greater extent than after the application without SA. This is substantiated by the results obtained both in the present and in previous studies conducted on tomato plants (Smoleń et al., 2015).

There is a need for conducting further studies with lower doses of both tested beneficial elements to levels that allow the balance of the daily diet of I and Se to reach the molar ratio of I:Se within the recommended RDA range of 4.4–8.8:1. This is also dictated by the need for obtaining lettuce yield not only characterized by higher accumulation of I and Se but also with unaffected content of other nutritionally important mineral elements, such as Fe, Zn and Cu.

## Author Contributions

SS: Leader of the project, co-author of the method of lettuce biofortification with iodine and selenium with additional application of salicylic acid, coordinator of experiments and laboratory analyses, author of the method of determination of ascorbic acid by capillary electrophoresis, author of the modification method of determination of: iodine (by ICP-OES) as well as SeMet, SeCys and proline by capillary electrophoresis, conducted the analysis of results and prepared the manuscript; IK: Co-author of the method of lettuce biofortification with iodine and selenium with additional application of salicylic acid, conducting the experiment with the lettuce cultivation, performing chemical analysis; help with preparing of the manuscript; MC: Project of the part of plant molecular study, performing of the molecular analysis of plant samples, help with preparing of the manuscript; MH: Help with performing of the chemical analysis of plant samples, help with preparing of the manuscript; KK: Performing of the molecular analysis of plant samples; WS: Supervision over research, help with preparing of the manuscript.

## Conflict of Interest Statement

The authors declare that the research was conducted in the absence of any commercial or financial relationships that could be construed as a potential conflict of interest.

## Footnotes

1 https://www.ncbi.nlm.nih.gov

2 https://www.idtdna.com
